# Sepsis-driven metabolic reprogramming shapes cancer immunotherapy efficacy, metastatic potential, and drug sensitivity

**DOI:** 10.3389/fimmu.2025.1642477

**Published:** 2025-09-01

**Authors:** Leyi Wang, Changdong Wu, Ming Hou, Zhiwei Li

**Affiliations:** ^1^ People’s Hospital of Xinjiang Uygur Autonomous Region, Urumqi, China; ^2^ Clinical Laboratory Center, People’s Hospital of Xinjiang Uygur Autonomous Region, Urumqi, China

**Keywords:** sepsis, cancer, metabolic reprogramming, immunotherapy, metastasis, biomarkers

## Abstract

Sepsis and cancer interact in a complex, bidirectional manner that significantly impacts patient prognosis, with metabolic reprogramming being a key factor. Sepsis-induced immune dysregulation and metabolic changes promote immunosuppression, tumor growth, metastasis, and resistance to immunotherapy. Cancer patients, especially those on immunosuppressive therapies, are more vulnerable to sepsis, complicating treatment and worsening outcomes. An integrated approach combining immunotherapy, metabolic interventions, and antimicrobial strategies is essential, alongside identifying biomarkers for personalized care. Recent advancements emphasize the need to integrate molecular insights, immunotherapy, and drug sensitivity analysis. This review explores how sepsis-driven metabolic reprogramming affects cancer immunotherapy and metastasis, providing a foundation for future integrated treatment strategies. Further research should focus on developing precise therapies that regulate metabolism, immunity, and the microbiome.

## Graphical Abstract

The interaction between sepsis and cancer presents significant clinical challenges, as illustrated in the graphical abstract. **(A)** Sepsis disrupts the tumor microenvironment by triggering inflammation and immune suppression, promoting tumor growth and immune escape through mechanisms like PD-L1 expression. **(B)** Sepsis alters tumor cell metabolism, increasing ROS production and metabolic stress, which accelerates tumor growth and complicates cancer treatment. **(C)** Sepsis in cancer patients often presents with atypical symptoms like fever and organ dysfunction, making early diagnosis difficult. Timely detection using biomarkers and advanced imaging is essential. **(D)** Effective treatment strategies involve combining immunotherapy, antibiotics, and antitumor drugs, with emerging therapies like engineered bacteria and exosome-based nanomedicine offering innovative dual-targeting approaches. Multidisciplinary collaboration and precision medicine are key to improving outcomes in this high-risk population.

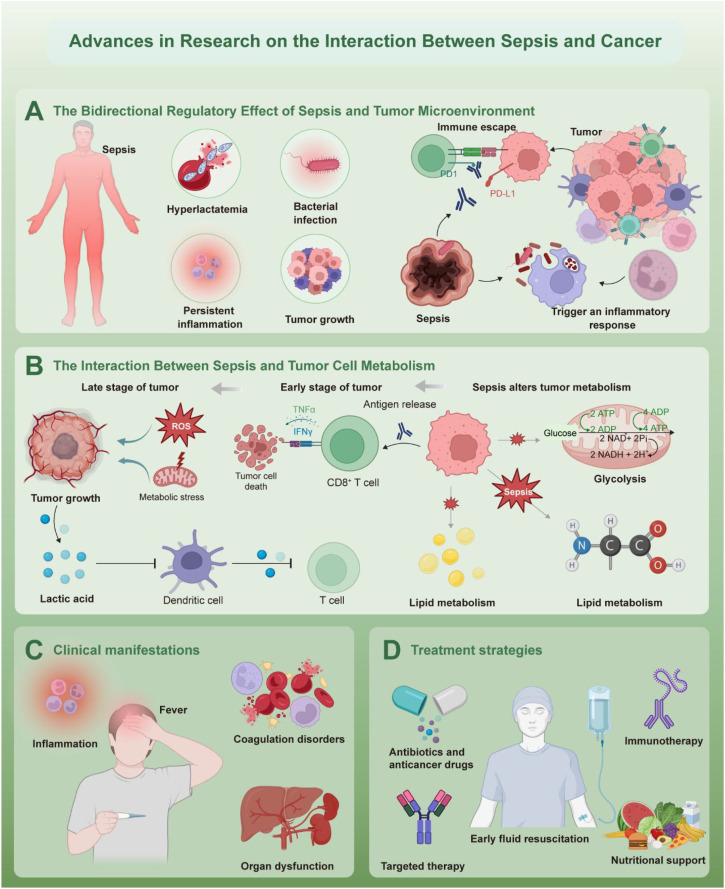

## 1 The basic theory of sepsis and tumor interaction

### 1.1 Pathological mechanisms and impact on the tumor microenvironment

Sepsis is a life-threatening condition characterized by a dysregulated immune response to infection, leading to systemic inflammation, immune dysfunction, and multiorgan failure (1). A hallmark is the cytokine storm, primarily driven by TNF-α, IL-1β, and IL-6, which induces endothelial damage, vascular leakage, coagulopathy, and subsequent immunosuppression through lymphocyte apoptosis and impaired antigen presentation (2). T cell exhaustion in the septic TME is characterized by elevated expression of inhibitory receptors (PD-1, CTLA-4, TIM-3, LAG-3) on CD8^+^ T cells, driven by chronic antigen exposure and sustained NF-κB/STAT3 signaling (3). Transcription factors such as TOX, TOX2, and NR4A family members maintain the epigenetic and transcriptional programs underlying exhaustion (4, 5). Pathogen-associated molecular patterns (PAMPs), such as LPS, activate Toll-like receptors (TLRs) on TME-resident cells, amplifying NF-κB/STAT3 signaling and promoting tumor survival and metastasis (3). Tumor cells contribute to systemic inflammation by secreting pro-inflammatory cytokines (e.g., IL-1β, IL-6) and chemokines (e.g., CCL2), which intensify immune dysregulation (6). Glioma-derived CCL2 and CCL7 facilitate monocytic-MDSC migration via CCR2^+^/CX3CR1^+^ signaling, reinforcing local immunosuppression (7). Sepsis induced extracellular matrix (ECM) remodeling, mediated by matrix metalloproteinases (MMPs), promotes tumor motility, invasiveness, and angiogenesis, facilitating dissemination (8). The interaction between sepsis and the tumor microenvironment (TME) is bidirectional; although acute infection may temporarily enhance anti-tumor immunity, chronic sepsis contributes to immunosuppression, metabolic reprogramming, and tissue remodeling. Recent studies have demonstrated that tumor-derived apoptotic extracellular vesicles promote metastasis and stemness through TME-mediated communication, unveiling a novel mechanism by which tumors exploit sepsis-induced alterations (9). This emerging paradigm warrants further mechanistic investigation.

### 1.2 Immunological basis of tumor associated sepsis

Cancer patients, especially those receiving cytotoxic therapies, face elevated sepsis risk due to therapy-induced immunosuppression (10). Sepsis exacerbates immune dysfunction and accelerates tumor progression, partly through the expansion of granulocytic myeloid-derived suppressor cells (G-MDSCs), which inhibit T cell responses via arginase-1, nitric oxide, and reactive oxygen species (11). In sepsis-induced tumor models, TLR4/MyD88 signaling boosts G-MDSC survival and function, promoting tumor growth. Sepsis also causes lymphopenia, T cell exhaustion (PD-1, TIM-3, LAG-3), and dendritic cell dysfunction, impairing tumor surveillance and reducing immunotherapy effectiveness (12, 13). Immune checkpoint inhibitor (ICI) resistance remains a major obstacle. Calreticulin-expressing liposomal nanoparticles (CRT-NPs) have reversed anti-CTLA-4 resistance in colon cancer by inducing immunogenic cell death, enhancing T cell activity, and reducing regulatory T cells (14). In gastric cancer, a low VEGF-A121/VEGF-A165 ratio was associated with improved progression-free survival, suggesting predictive value for ICI response. In clear cell renal cell carcinoma (ccRCC), resistance to combined anti-PD-1/CTLA-4 therapy was linked to myeloid-driven immunosuppression and alternative checkpoint pathways beyond PD-1 and CTLA-4 (15, 16). These findings highlight the multifactorial nature of immune resistance within the tumor–sepsis axis, emphasizing the need for personalized, targeted immunotherapies to overcome complex immunosuppressive networks.

### 1.3 Interaction between sepsis and tumor cell metabolism

Cancer cells exhibit metabolic reprogramming, notably the Warburg effect, characterized by elevated glycolysis despite sufficient oxygen availability, which supports cellular proliferation (17). Sepsis induces systemic metabolic disturbances, including hyperglycemia and insulin resistance, which affect both immune and tumor cell metabolism (18). In its early stages, sepsis enhances glycolysis and mitochondrial respiration in immune cells, augmenting anti-tumor activity. However, prolonged sepsis leads to metabolic exhaustion and immune dysfunction, fostering a tumor-permissive environment (19). Macrophage-targeted autophagy may mitigate this dysregulation by promoting inflammation resolution and tissue repair (20). Lactate, a glycolysis byproduct elevated in both cancer and sepsis, suppresses immune responses by impairing dendritic and T cell function and promoting Treg and MDSC expansion. Lactate also stabilizes Tregs via MOESIN lactylation, enhancing TGF-β/SMAD3 signaling and reinforcing immunosuppression.

**Figure 1:**
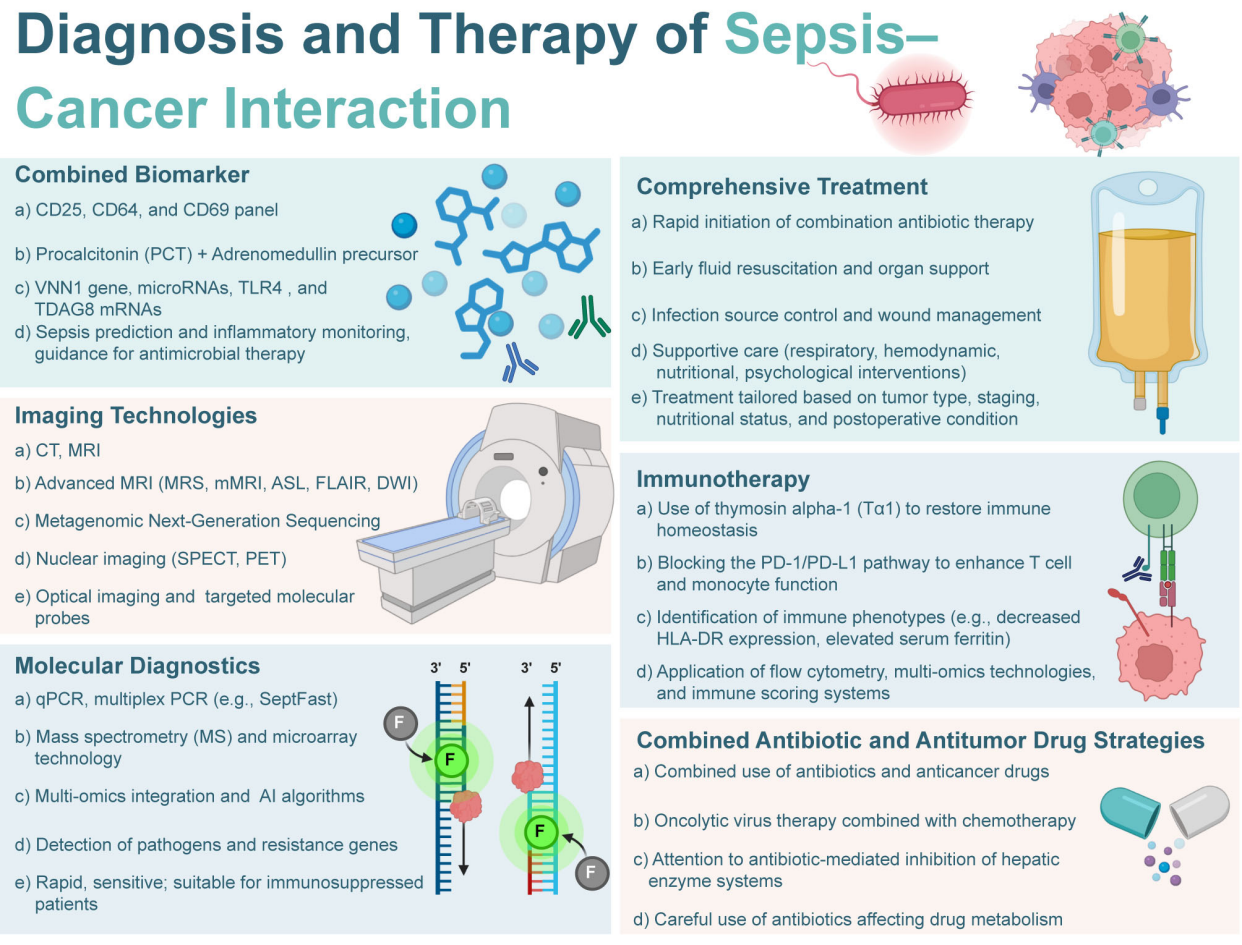
Diagnosis and therapy of sepsis-cancer interaction. The image provides an overview of various approaches and technologies used in the management of sepsis in cancer patients.

Sepsis-induced oxidative stress further supports tumor survival through DNA damage, activation of PI3K/Akt and MAPK pathways, and oncogenic transformation (21). The impact of sepsis on tumor progression is context-dependent. In murine models, polymicrobial sepsis inhibited tumor growth via TLR4-mediated NK cell activation, suggesting that sepsis-induced innate immunity can have anti-tumor effects under certain conditions (22). GLUT1 upregulation promotes glycolysis and NF-κB-driven inflammation in macrophages; inhibiting it impairs cytokine production and mitochondrial function, indicating similar reprogramming in tumor-associated macrophages (23). Moreover, the upregulation of CPT1A, linked to enhanced lipid metabolism and tumor suppression in colitis-associated cancer models, may be disrupted during sepsis, altering the tumor’s energy balance (24). This intricate metabolic crosstalk offers opportunities for therapeutic intervention, such as exercise-induced adipokine secretion, which regulates tumor metabolic reprogramming and may enhance immunotherapy efficacy when combined with exercise interventions under septic conditions (25–27) (**Figure 1**).

## 2 Epidemiology of sepsis and tumor interaction

### 2.1 Incidence and mortality of sepsis in cancer patients

Sepsis, a life threatening consequence of dysregulated host responses to infection, presents a major clinical challenge in oncology (28).Due to malignancy induced immunosuppression and aggressive anticancer therapies, cancer patients face a significantly higher risk of sepsis incidence and mortality compared to the general population (29). Contributing factors include tumor-induced immune evasion, disruption of anatomical barriers by invasive tumor growth, and immunosuppression resulting from cytotoxic therapies (30). Notably, despite their anti-inflammatory properties, statins did not confer a protective effect against sepsis in cancer patients, indicating complex immunopathological mechanisms beyond inflammation alone. Cancer stem cells further exacerbate immune evasion under septic conditions by enhancing metastatic potential and promoting drug resistance (31). Although sepsis-related mortality among cancer patients has declined in recent years—reflecting advances in oncologic care—certain subgroups remain disproportionately vulnerable (32). Hematologic malignancies, such as acute myeloid leukemia and multiple myeloma, confer particularly high sepsis risk due to severe neutropenia and bone marrow failure (33). The rising threat of antimicrobial resistance, especially infections caused by carbapenem-resistant Gram-negative bacteria, further complicates sepsis management, elevating mortality and constraining effective treatment options (34).

### 2.2 Impact of cancer type on sepsis incidence

Sepsis risk varies significantly by cancer type, tumor biology, and treatment modality (35). Solid tumors and hematologic malignancies exhibit distinct infection profiles (36). Recent Mendelian randomization studies have established a causal link between specific malignancies, particularly lung cancer (squamous cell carcinoma, adenocarcinoma, and small cell carcinoma), and increased sepsis susceptibility (37). This association is consistent with local tissue destruction, chronic inflammation, and opportunistic infections common in pulmonary cancers. In addition to tumor type, patient-specific factors such as advanced age, comorbidities, recurrent infections, and previous hospitalizations also contribute to an increased risk of sepsis (38). Anatomical patterns also emerge: solid tumors are often linked to intra-abdominal infections (e.g., post-surgical complications), while hematologic cancers predispose to bloodstream and pulmonary infections due to systemic immunosuppression (39). Iatrogenic factors, such as central venous catheters, chemotherapy-induced neutropenia, and mucosal barrier injury from radiotherapy, further exacerbate sepsis risk (40).

### 2.3 Correlation between sepsis and cancer patient prognosis

Beyond its acute mortality, sepsis exerts profound long-term effects on cancer outcomes, potentially accelerating tumor progression, promoting relapse, and reducing survival (41). Prognostic studies have identified key markers, including the neutrophil to lymphocyte ratio (NLR), brain natriuretic peptide (BNP), fluid balance, and SOFA scores, as predictors of short-term mortality in patients with sepsis and cancer (42).These markers offer promising avenues for early risk stratification and individualized management. However, the prognostic impact of sepsis is context dependent (43). For instance, in advanced epithelial ovarian cancer, sepsis did not significantly affect progression-free or overall survival, suggesting that host immune competence and tumor biology modulate outcomes (44). Notably, exercise-induced interleukin-15 (IL-15) has emerged as a potential prognostic biomarker in this setting, with serum levels positively correlating with tumor inhibition (45). This observation underscores the potential of exercise-mediated immune modulation as a therapeutic adjunct in septic oncology patients. Nonetheless, accumulating evidence supports the hypothesis that postoperative sepsis fosters tumor recurrence and worsens survival by inducing immune exhaustion and promoting tumor microenvironmental changes conducive to metastasis and angiogenesis (46). Clinically, these findings advocate for integrating sepsis prevention and rapid intervention into oncologic care protocols, emphasizing infection control, antimicrobial stewardship, and immune modulation (47).

## 3 Clinical manifestations of sepsis and tumor interaction

### 3.1 Typical clinical manifestations of sepsis in cancer patients

Sepsis, a life-threatening organ dysfunction from a dysregulated host response to infection, is particularly severe in cancer patients due to tumor- and treatment-induced immunosuppression (48). Compared to the general population, cancer patients exhibit higher sepsis incidence and 30-day mortality, driven by advanced disease, comorbidities, and immune dysfunction (49).A retrospective study of 435 ICU-admitted cancer patients identified metastatic disease, elevated serum lactate, and need for advanced life support as key mortality predictors, indicating severe physiological compromise (50). Classic signs of sepsis, including fever, leukocytosis or leukopenia, and coagulopathy, may be absent or atypical in immunocompromised patients, thereby complicating the diagnostic process (51). Hypothermia or afebrile presentations can obscure early signs, while viral endothelial injury exacerbates coagulopathy via platelet activation (52). Chronic tumor-related inflammation can further mask sepsis, requiring individualized assessment. Cancer therapies (chemotherapy, radiotherapy, targeted agents) impair immunity, while tumor invasion and surgeries disrupt barriers, facilitating infections. Infection patterns vary by cancer type: abdominal infections are common in solid tumors, and pulmonary and bloodstream infections are common in hematologic malignancies, often due to neutropenia (53). Cancer patients also face higher rates of hospital-acquired infections (HAIs), exacerbated by prolonged hospitalization, invasive procedures, and broad-spectrum antibiotics, leading to multidrug resistance (54, 55).

### 3.2 Diagnostic challenges of sepsis in cancer patients

Timely and accurate diagnosis of sepsis is critical in cancer patients, but oncologic care presents substantial diagnostic challenges (56). Traditional markers (e.g., CRP, PCT, leukocyte counts) have reduced sensitivity and specificity, particularly in those receiving chemotherapy or immunotherapy (57). The high prevalence of neutropenia and leukopenia further limits the diagnostic utility of conventional inflammatory parameters (58). Prior anticancer treatments further confound clinical assessment. Immunotherapy can trigger immune-related adverse events that mimic infection, while corticosteroids may suppress overt inflammatory signs (59). Additionally, paraneoplastic effects in certain cancers can distort biomarker levels, leading to false findings (60). High colonization rates, especially with indwelling devices or mucositis, increase the risk of culture contamination. These challenges underscore the need for cancer-specific diagnostic criteria for sepsis. Multifactorial strategies integrating tumor type, treatment history, immune status, infection risk, and organ function are essential (61). Combined biomarker panels (e.g., CRP, PCT, IL-6, sTREM-1) show promise, but require further validation (62). Machine learning and clinical decision support systems in electronic health records may improve early detection and risk assessment (63).

### 3.3 Analysis of sepsis related complications in cancer patients

Sepsis in cancer patients is associated with high rates of acute and chronic complications, contributing to poor short- and long-term outcomes (64). Multiple organ dysfunction syndrome (MODS), particularly post-surgery, is a major sequela (65). Surgical stress induces transient immunosuppression, increasing infection risk and potentially promoting tumor recurrence by impairing immune surveillance (66). Gram-negative and polymicrobial infections, often resulting from gut translocation or nosocomial sources, were prevalent, thereby complicating antimicrobial management (67). Infection profiles evolve with immunosuppression: bacterial pathogens dominate early neutropenic phases, while fungal infections (e.g., *Candida*, *Aspergillus*) emerge later (68). Antibiotic resistance and superinfections complicate therapy, necessitating a balance between empirical and targeted approaches (69). For instance, oral hydrogels may counter antibiotic-induced immunosuppression by modulating the gut microbiota–immune axis (70). The complex cancer–sepsis interplay demands individualized, multidisciplinary management (71). Collaboration among oncologists, infectious disease specialists, intensivists, and microbiologists is essential to improve outcomes (72).

## 4 Diagnostic technologies for the interaction between sepsis and cancer

### 4.1 Research on combined biomarkers for sepsis and cancer

**Figure 2:**
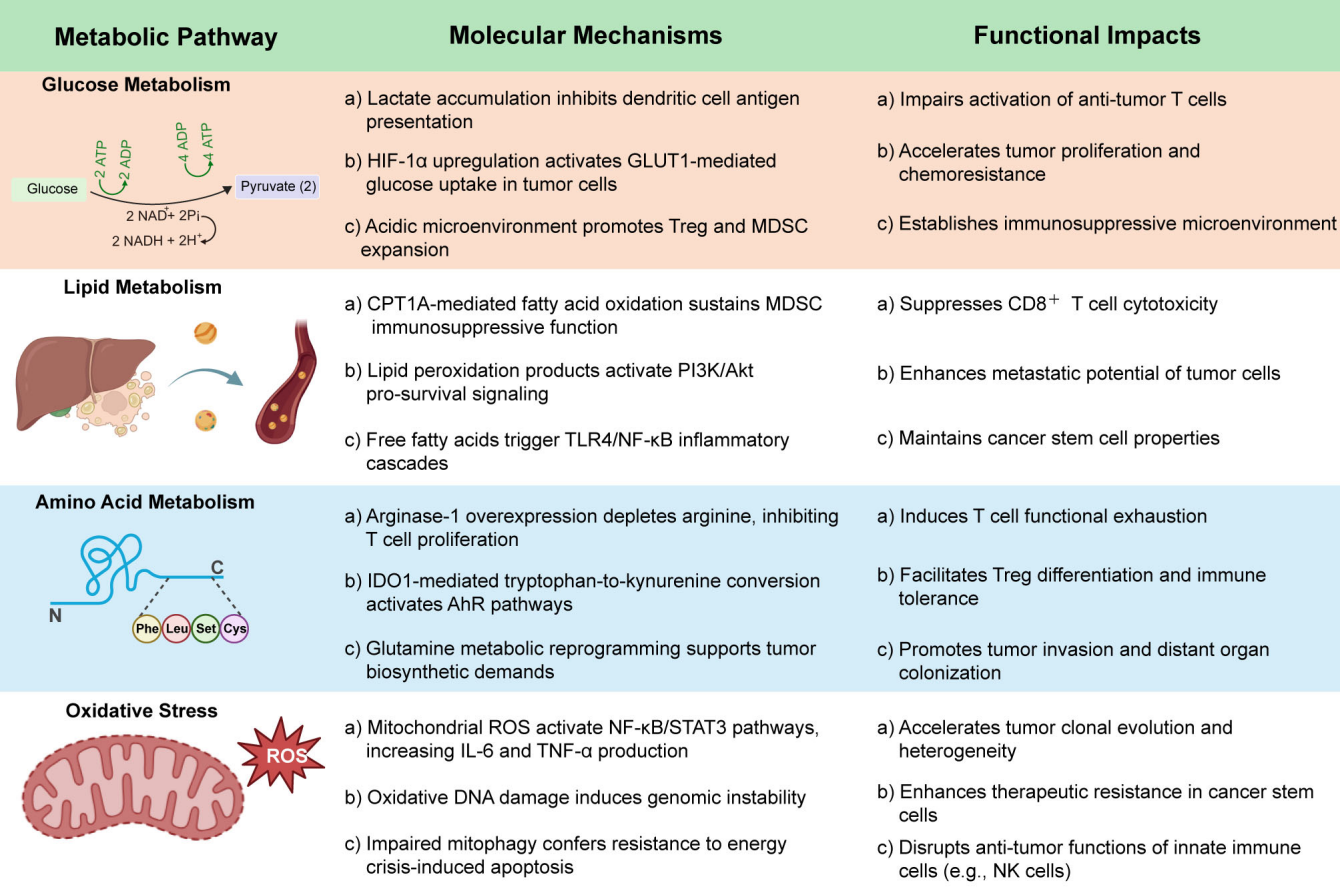
Sepsis-induced metabolic reprogramming in cancer immunosuppression and progression.

Diagnosing sepsis in cancer patients is challenging due to overlapping tumor-related inflammatory responses (73). Traditional biomarkers like CRP and PCT lack specificity in oncology settings due to tumor-associated factors (74). To address this, combined biomarker panels have been developed. For instance, a panel including CD25, CD64, and CD69 achieved an AUC of 0.978, outperforming individual markers (**Figure 2**) (75). At the molecular level, VNN1 and microRNAs (miR-146a, miR-155, miR-223) are promising biomarkers (76). VNN1, involved in oxidative stress and immune modulation, correlates with both sepsis and cancer outcomes (77). MicroRNAs help differentiate septic from tumor-related inflammation (78). Emerging multi-omics approaches, integrated with machine learning, are set to enhance diagnostic strategies and individualized treatment (79–81).

### 4.2 Application of imaging techniques in the diagnosis of sepsis and cancer

Imaging remains indispensable for the detection and management of sepsis and malignancy (79). Advanced magnetic resonance imaging (MRI) techniques, including magnetic resonance spectroscopy (MRS), molecular MRI (mMRI), arterial spin labeling (ASL), fluid-attenuated inversion recovery (FLAIR), and diffusion-weighted imaging (DWI), enable early detection of sepsis-associated encephalopathy (SAE), particularly in oncology patients whose neurological symptoms may be atypical or obscured by treatment effects (**Figure 1**) (82, 83). FLAIR and DWI are sensitive to white matter changes and cytotoxic edema, while MRS provides metabolic insights, facilitating timely neuroprotective interventions (84). Beyond neuroimaging, plasma metagenomic next-generation sequencing (mNGS) demonstrates superior pathogen detection compared to conventional blood cultures, which is critical in cancer patients with atypical infections (85). Advances in optical imaging, employing fluorescent and bioluminescent probes targeting bacterial structures, allow real time visualization of microbial burden, enhancing surgical and therapeutic decision making (86). Moreover, molecular diagnostics have markedly improved the sensitivity, specificity, and speed of infectious and oncologic disease detection (87).In sepsis, multi-omics approaches have identified transcriptomic, proteomic, and metabolomic signatures that define distinct disease subtypes and prognostic trajectories, promoting a shift toward precision diagnostics (88). PCR based technologies, including multiplex assays like SeptFast, allow rapid, simultaneous pathogen and resistance gene detection, significantly reducing time to treatment despite challenges such as contamination risk and limited detection of novel organisms(**Figure 2**) (89). Meanwhile, microarrays and nanotechnology based biosensors facilitate high throughput, point of care testing, critical for intensive care and oncology settings (90–92).The integration of multi-omics data with artificial intelligence (AI) and machine learning is an emerging frontier (93). Predictive models based on large datasets can uncover complex disease patterns, forecast therapeutic responses, and guide individualized treatment plans, greatly enhancing clinical decision making in patients navigating both cancer and sepsis (94).

## 5 Sepsis and cancer: integrated treatment strategies and future perspectives

### 5.1 Multidisciplinary approaches for managing sepsis in cancer patients

The coexistence of sepsis and cancer presents a major clinical challenge, requiring dynamic management strategies to address immune, metabolic, and physiological disruptions (95). Treatment must focus on infection control, tumor progression, immune stabilization, and preserving physiological function. Early intervention is critical, as delays in antibiotics worsen outcomes (**Figure 2**) (96). Multifunctional composite hydrogels offer synergistic benefits for sepsis in bone metastasis, combining immunomodulatory and osteogenic effects (97). Supportive therapies, including fluid resuscitation, hemodynamic management, respiratory support, nutrition, and psychological care, should be tailored to individual patient needs. A multidisciplinary team of intensivists, oncologists, and other specialists is essential for optimizing patient survival and care (98).

### 5.2 Emerging immunotherapeutic and pharmacological innovations

Advances in immunotherapy have opened new avenues for addressing the immune dysregulation inherent in both sepsis and cancer (**Figure 1**) (99). Non-invasive physical stimulation can enhance immune cell infiltration and mitigate T-cell depletion by altering the tumor microenvironment (100). Agents such as thymosin α-1 improve immune competence and survival in sepsis, while immune checkpoint inhibitors (ICIs) reverse T-cell exhaustion and enhance antigen presentation (101). Preclinical data indicate that anti-PD-1/PD-L1 antibodies function through Fcγ receptor-dependent mechanisms, although FcγR-independent variants may be more effective in immunocompromised settings, underscoring the need to optimize ICI formats (102). Immunotherapies also influence immune cell metabolism, particularly glycolysis and mitochondrial oxidative phosphorylation, which are critical during sepsis. Given the heterogeneity of immune status in sepsis, biomarkers like mHLA-DR and ferritin can help identify candidates for immunometabolic therapy (103). T cell therapies targeting oncogenic viral antigens may overcome antigen presentation deficits induced by sepsis, with efficacy enhanced by metabolic modulation, such as reducing lactate and promoting fatty acid oxidation. CD4^+^ T cells in sepsis exhibit persistent mitochondrial dysfunction and elevated glycolysis, leading to IL-17 dysregulation and impaired secondary responses (104). Targeting these pathways may restore T cell function post-sepsis. Novel delivery platforms, such as engineered bacteria, bacterial vesicles, and exosome-based systems, enable targeted delivery of metabolic modulators (e.g., NAD^+^ precursors, AMPK activators, and short-chain fatty acids) to immunosuppressive sites. These strategies address mitochondrial dysfunction and energy depletion characteristic of late-stage sepsis (105–109).

### 5.3 Personalization of therapy and optimization of drug interactions

Polypharmacy in septic cancer patients significantly increases the risk of drug–drug interactions, particularly through the modulation of cytochrome P450 enzymes by antibiotics and antifungals, thereby altering the pharmacokinetics and toxicity profiles of chemotherapeutic agents (110). To address these complexities, strategies such as therapeutic drug monitoring, pharmacogenetic testing, computer-aided drug design techniques, and AI-driven clinical decision support systems are needed (**Figure 1**) (111). Moreover, the transition toward personalized medicine, guided by immunophenotyping, transcriptomics, and dynamic biomarker monitoring, holds considerable promise for both sepsis and oncology care (112).Tailored treatment plans that incorporate genetic, metabolic, and immune profiles may enhance therapeutic efficacy while minimizing adverse effects, particularly in vulnerable patient populations. Prospective validation of precision-based protocols is crucial to fully realize the potential of individualized therapy in improving outcomes for patients confronting the dual burden of sepsis and malignancy.

## 6 Future perspectives on the interaction between sepsis and cancer

Advances in biomedical engineering, systems biology, and molecular diagnostics have propelled sepsis and cancer research (113). Technologies like high-resolution biosensors improve early detection, while CRISPR/Cas9 aids targeted drug discovery (114). Next-generation sequencing enhances pathogen identification, and AI revolutionizes risk stratification in oncologic sepsis (115, 116). These innovations drive precision diagnostics and individualized treatments. Therapeutic targets such as inflammatory caspases, PDGFB, TLR4, and PD-1/PD-L1 are being explored for their roles in immune modulation and tumor growth (117). However, ethical issues arise, particularly around consent in acute sepsis (118) and concerns over patient privacy with AI and big data (119). Addressing these is vital for equitable healthcare (120).

## 7 Conclusion

In conclusion, the bidirectional relationship between sepsis and cancer complicates patient management, as sepsis-induced immune dysfunction and metabolic reprogramming hinder cancer progression, impair immunotherapy, and promote metastasis. These metabolic shifts in cancer and immune cells worsen the disease, resulting in a more aggressive clinical course. To improve outcomes, integrated therapies addressing both immune and metabolic disturbances are essential. Combining immunotherapy, metabolic interventions, and antimicrobial treatments, along with identifying relevant biomarkers, could significantly enhance personalized cancer care. Ongoing research is expected to lead to better management strategies and improved prognosis for cancer patients at risk of sepsis.
